# Towards large scale automated cage monitoring – Diurnal rhythm and impact of interventions on in-cage activity of C57BL/6J mice recorded 24/7 with a non-disrupting capacitive-based technique

**DOI:** 10.1371/journal.pone.0211063

**Published:** 2019-02-04

**Authors:** Karin Pernold, F. Iannello, B. E. Low, M. Rigamonti, G. Rosati, F. Scavizzi, J. Wang, M. Raspa, M. V. Wiles, B. Ulfhake

**Affiliations:** 1 Departments of Neuroscience, Karolinska Institutet, Stockholm, Sweden; 2 Tecniplast SpA, Buguggiate (Va), Italy; 3 The Jackson Laboratory, Bar Harbor, Maine, United States of America; 4 National Research Council, CNR-Campus International Development (EMMA-INFRAFRONTIER-IMPC), Monterotondo Scalo, Rome, Italy; Kent State University, UNITED STATES

## Abstract

**Background and aims:**

Automated recording of laboratory animal’s home cage behavior is receiving increasing attention since such non-intruding surveillance will aid in the unbiased understanding of animal cage behavior potentially improving animal experimental reproducibility.

**Material and methods:**

Here we investigate activity of group held female C57BL/6J mice (*mus musculus*) housed in standard Individually Ventilated Cages across three test-sites: Consiglio Nazionale delle Ricerche (CNR, Rome, Italy), The Jackson Laboratory (JAX, Bar Harbor, USA) and Karolinska Insititutet (KI, Stockholm, Sweden). Additionally, comparison of female and male C57BL/6J mice was done at KI. Activity was recorded using a capacitive-based sensor placed non-intrusively on the cage rack under the home cage collecting activity data every 250 msec, 24/7. The data collection was analyzed using non-parametric analysis of variance for longitudinal data comparing sites, weekdays and sex.

**Results:**

The system detected an increase in activity preceding and peaking around lights-on followed by a decrease to a rest pattern. At lights off, activity increased substantially displaying a distinct temporal variation across this period. We also documented impact on mouse activity that standard animal handling procedures have, e.g. cage-changes, and show that such procedures are stressors impacting in-cage activity.

These key observations replicated across the three test-sites, however, it is also clear that, apparently minor local environmental differences generate significant behavioral variances between the sites and within sites across weeks. Comparison of gender revealed differences in activity in the response to cage-change lasting for days in male but not female mice; and apparently also impacting the response to other events such as lights-on in males. Females but not males showed a larger tendency for week-to-week variance in activity possibly reflecting estrous cycling.

**Conclusions:**

These data demonstrate that home cage monitoring is scalable and run in real time, providing complementary information for animal welfare measures, experimental design and phenotype characterization.

## Introduction

The use of animals in scientific experiments has many key advantages taking into consideration as it does, the complexity of the complete living organism however, there are also intrinsic challenges including a moral imperative to follow a “Replace, Reduce and Refine” philosophy [1,2], and to maximize data acquisition and usefulness in all experimental systems involving them. An absolute requirement for any experimental system is reproducibility [3]. Mice, especially inbred mice, form the cornerstone of a highly tractable mammalian experimental system however, living organisms including mice are highly sophisticated biological organisms showing a strong capability to react and adapt to the conditions they find themselves in. Thus, for ethical and scientific reasons the use of animal as experimental models, their characterization plus the provision of the best possible husbandry are prerequisites in optimizing their use and improving reproducibility. Over the past years, animal testing in academic discovery research has been the subject of substantial critique (e.g. [4–6]). Common flaws include insufficient reporting of animal strain used, husbandry practices, protocol details applied, design errors (randomization, bloc design and blinding) and lack of statistical power (idem; [7,8]). Further, animals, including mice display a rich repertoire of behavioral responses to experimental testing however despite our insights, it is surprisingly rare that these responses are recorded unless they are directly the subject of the study and reported as a read-out parameter (see also below), while in contrast for human clinical trials the complete collection of all data from patients, if only for compliance is a key requisite.

Automated home-cage monitoring ([9–15] for review see [6,16]) techniques are still in their infancy and are only beginning to attract the attention they deserve. Such systems, especially when applied large scale, hold the promise to advance mouse behavioral surveillance, providing essential data to assess and improve animal conditions (e.g. [17] [18]), breeding performance, evaluate phenotype deviation between and within strains (e.g.[15,16,19]), responses to animal procedures (this paper) 24/7, and poorly defined environmental impacts (idem). Crucially, all without handling the animals or placing them into a novel environment (test or examination area) (e. g [20,21]. The creation of such data lends itself to improved animal welfare and assists in the implementation of humane endpoints, and crucially it also generates mass data about home cage-life. Combined these data will lead towards improved experimental reproducibility.

Here we have implemented a 24/7, scalable activity monitoring system, based on recording perturbations in capacitance measurements. The sensors used are placed external and under the cage and reflect animal movement/motion across the home cage floor. The data generated allows us to explore if such a system can provide accurate meaningful surveillance of cage-life, leading to improving animal welfare measures and experimental/testing study design and analyses.

## Material and methods

**Fig 1:**
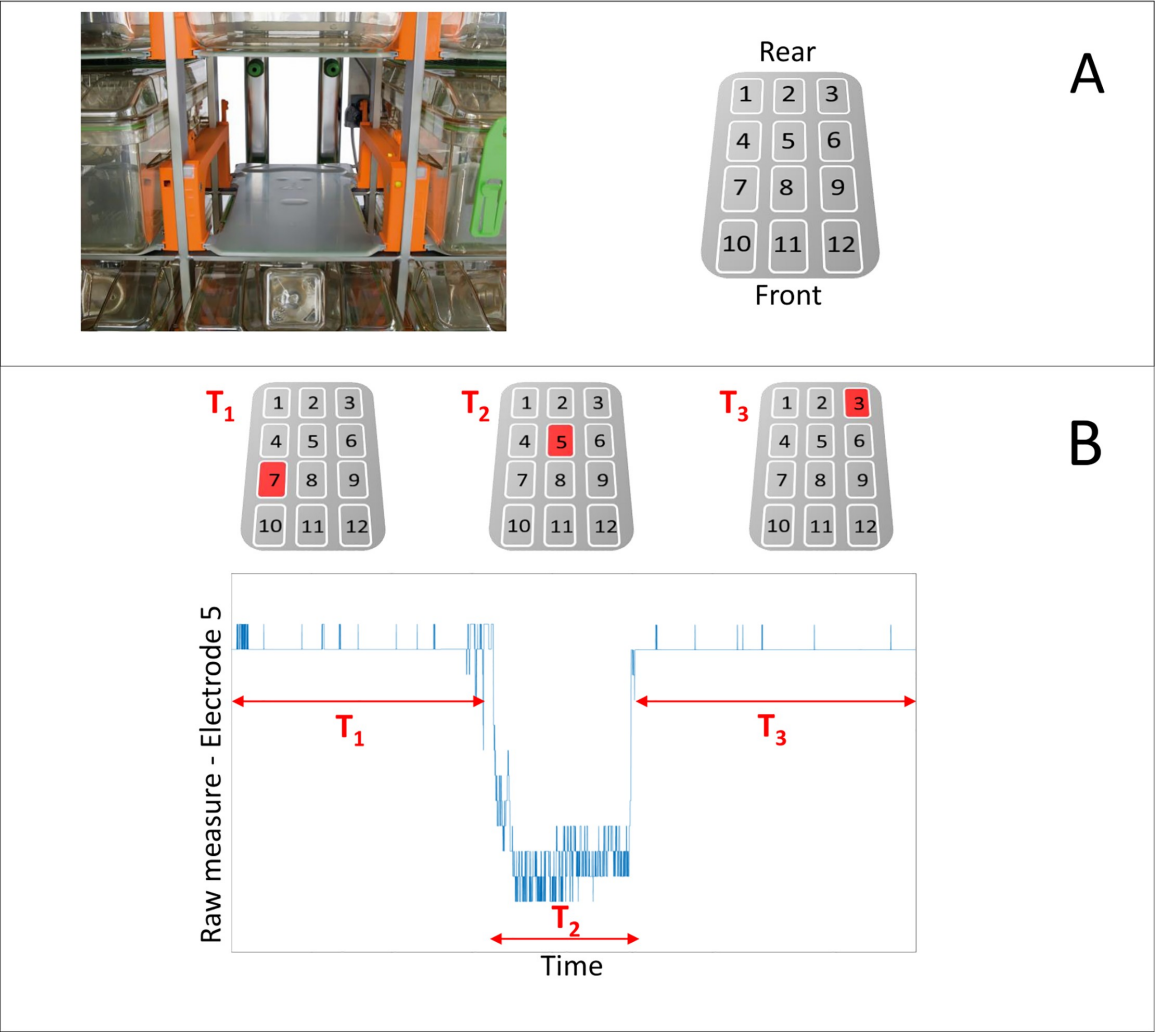
System description. A Standard IVC cage position equipped with DVC sensing technologies. The grey board contains the 12 electrodes, while the orange runners have embedded IR sensors for food and water bottle detection. Electrodes numbering is shown in the right panel; B shows example of raw signal measured from electrode 5 when a mouse stays for T_1_ seconds in a cage area close to electrode 7, then moves towards and across electrode 5 during T_2_ seconds and then moves on to electrode 3 staying there for T_3_ seconds.

**Fig 2:**
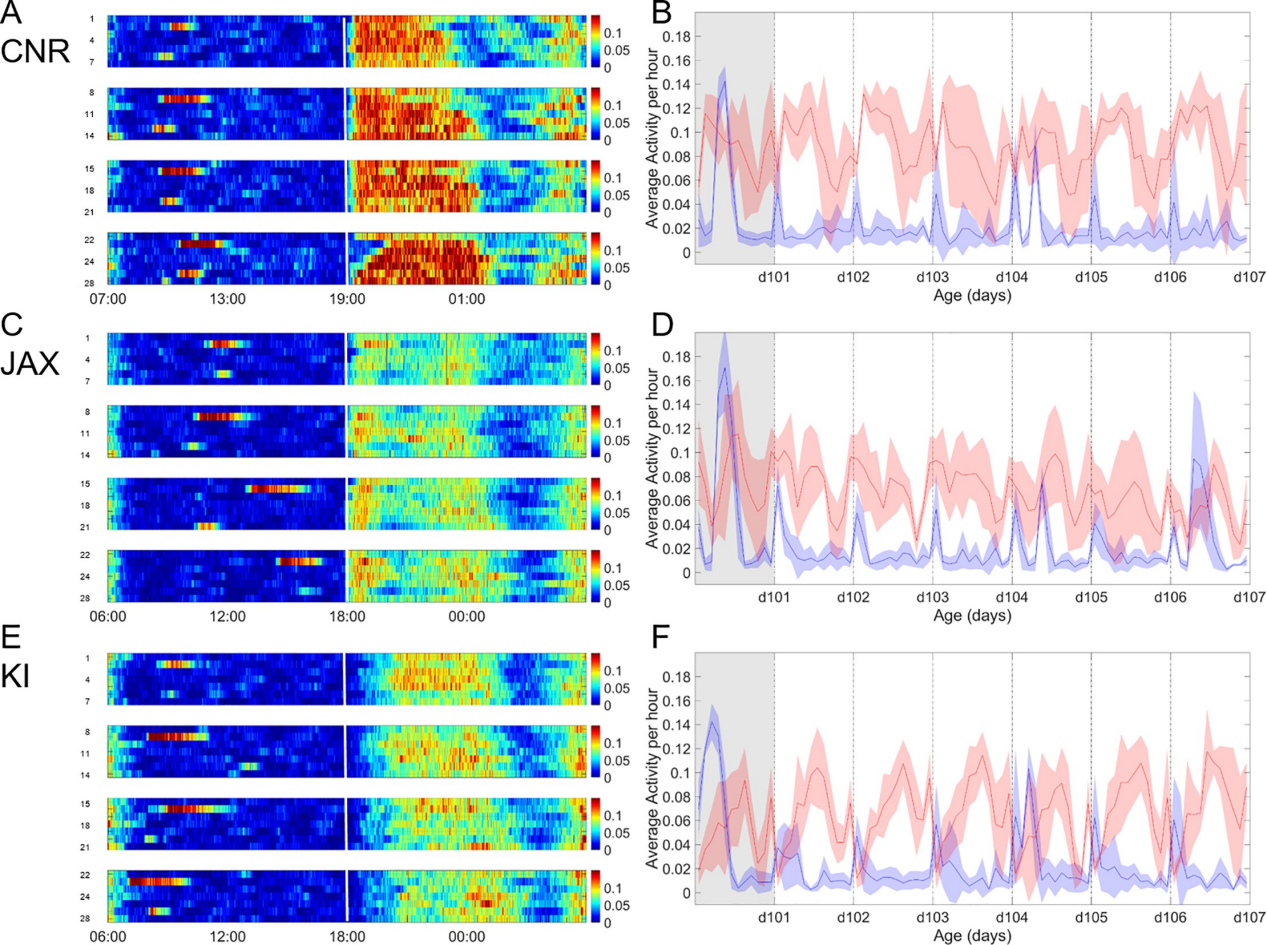
Day and night time activity at CNR, JAX and KI. A, C, E. Heat maps showing average global activity (all 12 electrodes) recorded from 5 cages with 5 female C57BL/6J in each cage, during 4 consecutive weeks (day 1 through 28 on the ordinate) at CNR (A), JAX (C) and KI (E); activity coded in color according to scale on the right side of the panels A, C and D. Each panel is 1 week; with days as rows on the ordinate and time h on the abscissa. To the left is day break with lights-on and white vertical line indicates transition to night time with lights-off. Cages were subjected to daily check-up which are done without removing the cage from the rack. Two more intrusive procedures are conducted weekly with a cage-change on Thursday and a second handling for weighing with health assessment on Monday; both procedures require cage removal from the rack, transferring to a changing station, opening of cage and removing animals from the cage; i.e. manual handling of each animal. The general day and night rhythm of activity alterations as well as the impact of procedures replicates well across sites. A main difference between sites was a generally higher level of activity (p<0.001) after at CNR (A) compared with both JAX (C) and KI (D) and also a slower increase in activity at lights-off at KI (cf. A-C-E and see also Figs 5 and 9). Note also that the response to lights-on starts prior to the event and last for an hour following lights-on while the response to lights-off occurs after darkness. Use of 30 minutes of dawn and dusk at light-on/off, i.e. slow light change transition, did not significantly alter the responses (not shown). B, D, F Actigrams showing global (all 12 electrodes) average activity per hour of the cages shown as heat maps in A, C and E (day 15 to 22). Each bin indicated by dotted vertical lines display the night time (12 hrs. of lights off) activity superimposed on day time activity (12 hrs. of lights on). The grey-shaded bin is day of cage-change. Abscissa denotes age of animals (in days) and ordinate average activity. The blue trace is average per h (of activity per minute) daytime activity (each bin starts at lights-on until lights-off) and shaded blue areas denotes the standard deviation. The red trace is the corresponding night time activity (each bin starts at lights-off until lights-on) i.e. with a 12 hrs. phase shift to the red traces, with the standard deviation indicated as shaded red area. As evident from the heat map and plots there is a significant difference between activity during day and night time at There is also a difference in activity between sites day and night (p<0.001).

The digital individually ventilated cage system (DVC^TM^) used here is more completely described in supporting information (S1 File) to this paper. Briefly, the core of the system is an electronic sensor board installed externally and below each standard IVC cage of a rack. The sensor board is composed of an array of 12 capacitive-based planar sensing electrodes (Fig 1A). A proximity sensor measures the electrical capacitance of each of the 12 electrodes 4 times per second (every 250ms). Their electrical capacitance is influenced by the dielectric properties of matter in close proximity to the electrode, leading to measurable capacitance changes due to the presence/movement of animals in the cage. Thus, movements across the electrode array are detected and recorded as alterations in capacitance (Fig 1B). By applying custom designed algorithms (S1 File) to the collected data we can infer information regarding in-cage animal activity (Fig 2). For this study, we used the first-order difference of the raw signal (i.e., capacitance measured every 250ms) as the basic metric of animal activity. More specifically, we take the absolute value of the difference between two successive measurements for each electrode (signals spaced 250 msec apart) and compare it against a set threshold (capacitance variations due to noise) to define an activity event. This metric thus considers any animal movement that generates a significant alteration in capacitance, an activity event (for further details see S1 File). Note, this activity metric represents the overall in-cage activity generated by all mice in a cage from any electrode and is not tracking activity of individual group-housed animals.

We used cohorts of C57BL/6J supplied by Jackson laboratories (JAX) and distributed either by JAX (all female mice derive from the same breeding colony at Bar Harbor, Maine) or their European partner Charles River Laboratories (CR; all males were from CR Germany and originating from JAX). Both male and female mice were deployed at an age of 6–8 weeks and recordings continued up until 25 weeks of age (Table 1). Upon arrival mice were setup blind into the DVC^TM^ system using Tecniplast GM500 cages (floor area approximately 500 cm^2^) with standard bedding (Table 2), food (SDS, RM3; radiated), and cage enrichments (see Tables 2 and 3). Vivarium light cycle was instant 12 hrs. on, 12 hrs. off/day. Cages were populated with 5 mice/cage; if a change in number of animals in a cage occurred, the cage was removed from the study. To examine the impact of different locations on reproducibility, recordings were conducted approximately in parallel at: Consiglio Nazionale delle Ricerche (CNR, Italy), The Jackson Laboratory (JAX, USA) and at Comparative medicine, the Karolinska Institutet (KI, Sweden) according to an agreed protocol (Tables 1–3 and S1 File)and the data compared. With the exception of the multicenter study, all mice recorded here were kept at the facility for other purposes and recorded from while they were awaiting testing.

**Table 1:** Table of animals used in the different recordings analyzed.

Cohorts	Strain^#^	Gender	Age*	# Cages	# animals
Multi-center study	C57BL/6J	Female	(6)8-25 weeks	15	75
CC M 1	C57BL/6J	Male	8–16 weeks	8	40

^#^All animals were either shipped from JAX USA (females) or through their European partner Charles River Laboratories (males).

* Range is age at deployment to end of recordings in weeks.

**Table 2:** Cage content and vivarium parameters.

Sub study	Bedding^#^	Food	Enrichment	Humidity	Temperature	Dark/light
KI—Multi-center study,	Corn cob	SDS RM3	House*, nesting towels	40%-60%	19–23 ^o^C	12/12
CNR—Multi-center study	Corn cob	SDS RM3	House*, nesting towels	40%-60%	19–23 ^o^C	12/12
JAX—Multi-center study	Corn cob	LabDiet 5k52 (6% fat)	House*, nesting towels	35%-45%	22–23 ^o^C	12/12
KI–CCM1	Aspen chips	SDS RM3	House*, Sizzel nest	40%-60%	19–23 ^o^C	12/12

^#^ Bedding was 100 g of Aspen chips or 200 g of Corn cob (large size).

*Mouse House a Tecniplast acre010-20cs.

**Table 3:** Vivarium parameters continued.

Site	Size of holding unit*	Light level center of room (day time, lux)	Light level at front of cages (day time, lux)	Light level night time	Number of staff/researcher trafficking the holding room
KI	15/512	200 (400)	20–30^§^ 95–134^#^	No white light, occasionally >620nm	1–7, mainly day time
JAX	15/1755	300 (500)	10–100	No light	2–6, only day time
CNR	15/15	250	72–230	No light, or occasionally white light at the changing station only	2–3; only day time

*Number of cages in the study/total number of cage-slots in the room.

^§^ Normal day time room light and changing station light when no work in the holding room is conducted.

^#^ Day time room light while animals are attended to by staff or researchers.

Numbers within brackets indicate maximum values in the room.

The basic entities used for analysis are site, sex, cage and day. Time-windows are either day (24h), night (lights-off, 12h), day time (lights-on, 12h) or week, the latter corresponding to a cage-change cycle, i.e. time between two successive cage-changes. Other time windows and entities are defined in the running text as they appear. Data are presented as activations recorded across electrodes (global activity) during a time-window; *normalized activity* is the activations recorded divided by the average activity during a defined time-window such as a cage-change cycle and, finally, as the *fraction of activity*, i.e. the percentage of the total activity allocated to a certain area or time-window (e.g. day and night). To delineate a change in activations occurring as a response to an event/procedure/intervention from base-line activity variations, the full-width half-maximum (FWHM) paradigm was used (for details on how this was computed, see S1 Fig and S1 File). Data are shown either as heat maps with color-coding, plots with continuous data presented as average with a confidence interval (95%). Box plots used show median, 25- and 75% quartiles and outliers.

Data were processed through scripts generated in either R, MATLAB^TM^ or Python^TM^. We used the rank-based analysis of variance-type statistic (ATS) to test differences across sites or sexes, weeks and days [22]. This is a non-parametric test for longitudinal data that does not require strong assumptions as the Repeated Measures ANOVA (which we found violated in some cases). Analyses were implemented using the nparLD package in R statistical software [23]. We considered cages as subjects, weeks and days post bedding change as within-subject factors (“sub-plot” repeated factors), site and sex as between-subject factors (“whole-plot” factors). According to authors’ terminology [23], we used four different tests in the paper: LD-F1 (one repeated factor: week), LD-F2 (two repeated factors: week and days), F1-LD-F1 (site or sex as the whole-plot factor and week as the repeated one), F1-LD-F2 (site or sex as the whole-plot factor and weeks and days as the repeated ones).

For all three test sites experimental procedures were agreed upon, reviewed and approved by local animals welfare oversight bodies: CNR, the experiments were performed with the approval and direct supervision of the CNR-IBCN/Infrafrontier—Animal Welfare and Ethical Review Body (AWERB), in accordance with general guidelines regarding animal experimentation, approved by the Italian Ministry of Health, in compliance with the Legislative Decree 26/2014 (ref.892/2017-PR), transposing the 2010/63/EU Directive on protection of animals used in research; JAX, the Institutional Animal Care and Use Committee (IACUC); KI by the Regional Ethics Council (Stockholms Djurförsöksetiska nämnd; project license N116-15). For further information of the protocol used in the multicenter study see supporting information.

## Results

### General day and night activity pattern observed in a DVC system

**Fig 3:**
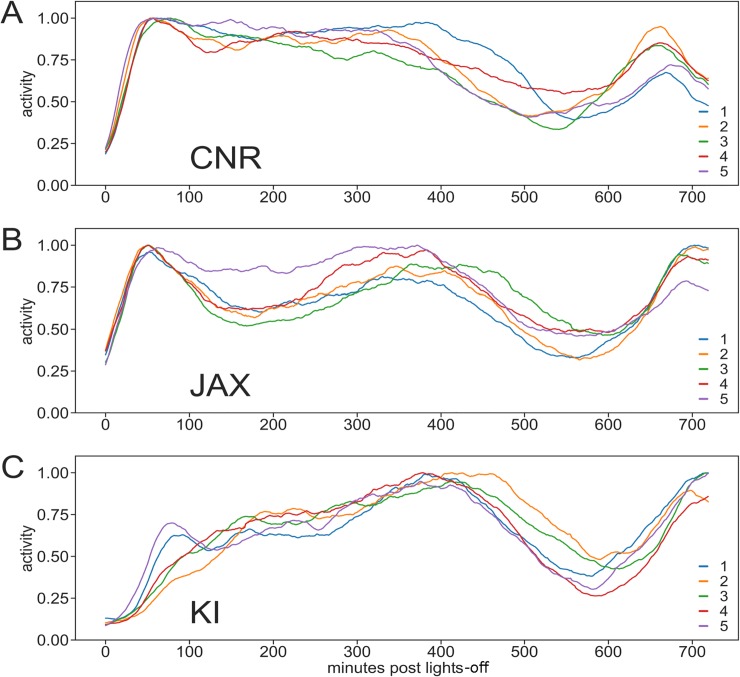
Activity following lights-off at CNR, JAX and KI. A-C Average response per cage across weeks to lights-off in cages (n = 5; individual traces in color) of female mice housed at the three sites. Traces start at light-off (abscissa, minutes) and ends at lights on (dark-cycle is 720 minutes). Each trace corresponds to one cage and is the average activity across multiple weeks normalized to peak activity (= 1.0). The plots deriving from the different sites show clear differences with a short time-to-peak latency at CNR (Table 4), while mice at JAX (B; Table 4) show a bi-phasic response with an early short latency time-to peak followed by a decrease in activity and a second peak at about 400 minutes. At KI (C, Table 4) there is more protracted onset of activity and the time to peak matches the second peak observed at JAX, i.e. 400 minutes after lights-off. The late-night pattern of activity, >400 minutes, show less difference across sites (see also Fig 8).

**Fig 4:**
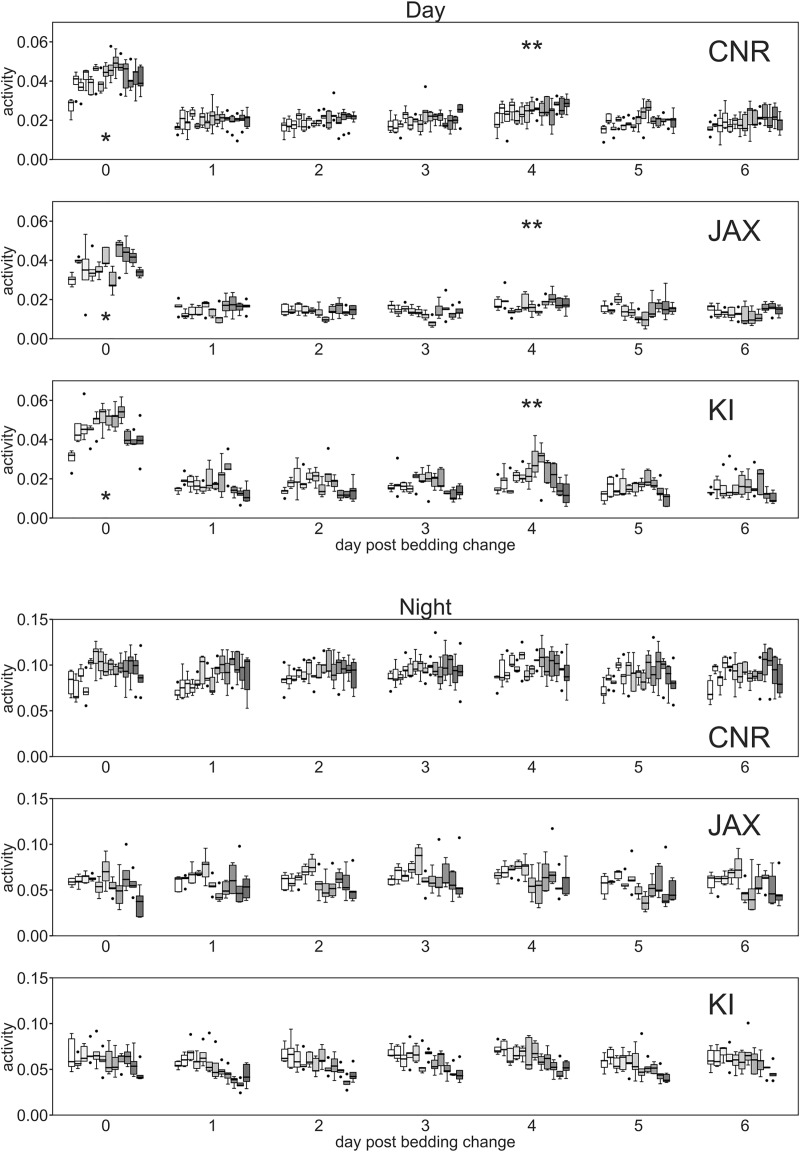
Day and night time activity at CNR, JAX and KI. Box plots (cages) showing global activity (ordinate) of the five cages during day time (A, D, G) and night-time (B, E, H) across **11–13** weeks for each day of the cage-change cycle (abscissa; 0 being day of cage-change). Weeks at each site are presented separately for each day of the cage-change cycle. Asterisk denote day of cage-change while double asterisk indicates day of weighing. At all three sites there were significant variation in day-time activity between weekdays (p<0.001) and also between weeks at each site (p<0.02–0.001), however, the week-to-week variation in activity did not co-vary with the variation of activity between weekday (weekday*week p>0.05).

As expected, mouse cage-life followed a distinct day and night (diurnal) pattern of activity alterations with 12 h of light and 12 h night (Figs 2–4) (see [24,25] for review [16] and references therein). Transition to night with (instant) lights-off stimulated a gradual increase in activity peaking within ~2 h (Figs 2 and 3, Table 4) followed by a variable high level of activity that continues for another 4–5 h (*idem*). Six to eight hours after lights-off there is a marked decrease in recorded activity moving towards a resting pattern until ~1–2 h before “day break” (light instant on), when animals become again quite active (Figs 2 and 4); a final burst of activity lasts ~1 h after lights-on (*idem*) and then reduces to a rest state (idem). During day-time mice rest and activity is low across the whole floor area, when the mice are left undisturbed (Fig 2 and S2 Fig). Thus, we conclude that the transition from day-to-night and night-to-day respectively, generate distinctly different responses and that late night (see also below) and all of the day time are periods for rest when group held animals are left undisturbed.

**Table 4:** Response to lights off.

Site	Average time to peak (min)	Average time to 90% percentile of peak (min)	Average peak (activations)	Average peak 90% percentile (activations)
CNR	346.8	120.6	0.1666	0.1420
JAX	361.2	124.3	0.1139	0.0936
KI	439.8	252.4	0.1245	0.0991

**Fig 5:**
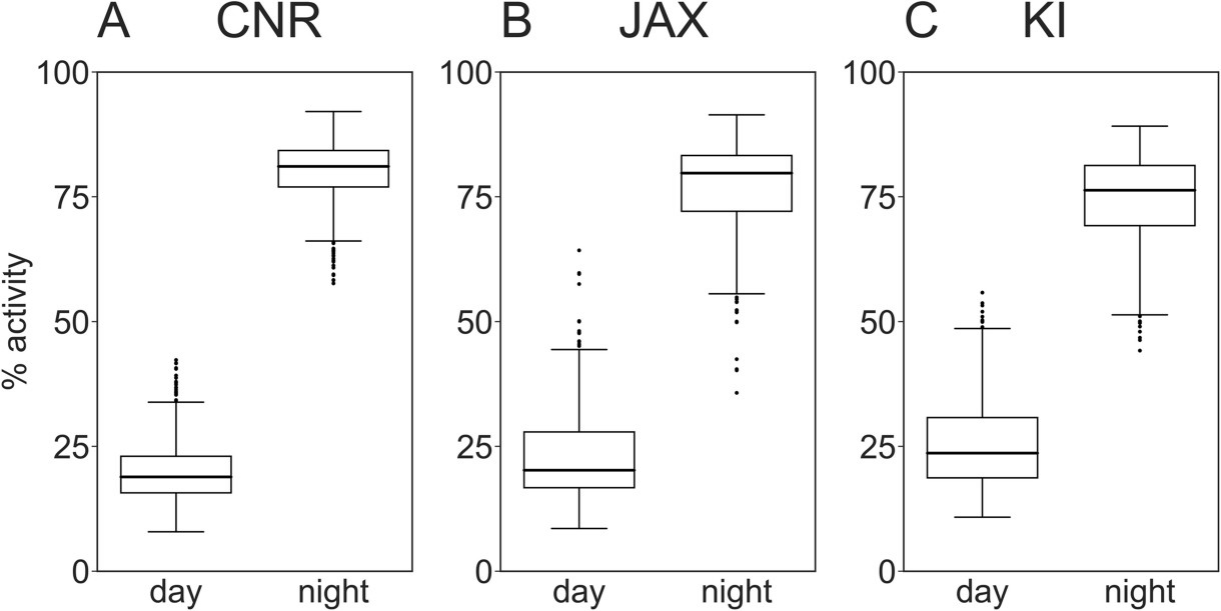
Fraction of total (24h) activity occurring during day time and night time, respectively, at CNR, JAX and KI. A, B and C show fraction (%) of total day (24 h) activity occurring during lights-on and lights-off, respectively, at the three sites. The distribution of activity between day and night time is highly significant (p<0.001) and quite similar across sites.

These tests were repeated at the three sites (Figs 2–4) using female C57BL/6J (see Tables 1–3 and S1 File for details on housing protocol). Data gathered clearly illustrate that the general pattern of activity variation with lights-on and—off, as well as re-occurring variation in activity during the lights-off period replicates across sites (idem). All three sites show similar significant effects of lights-on/lights-off and week day (Fig 4, effect of light factor p<0.001, effect of weekday p<0.02–0.001). The effect of weekday is instigated by cage-change day 0 and weighing of the animals on day 4 (Fig 4). Although a similar general pattern was observed, closer analysis of these data highlighted some significant differences in mouse cage activity between sites, week day and weeks (site factor and week factor both p<0.0001; day factor p<0.01–0.001) with a generally higher level of activity at CNR (see Discussion), and a smaller yet statistically significant (p<0.001) difference between JAX and KI. The distribution of activity between day and night (Fig 5) calculated as fraction of total activity across 24 h was similar across sites.

Within sites we also noted a significant variability between weeks and weekdays in the day and night time activity, respectively (Fig 4; effect of week p<0.02–0.001, effect of weekday p<0.01–0.001). However, if weekdays with procedures, e.g. cage change were omitted from the analyses the variance between days during lights-on was smaller and insignificant at CNR and JAX but still significant at KI (KI, p = 0.01).

**Fig 6:**
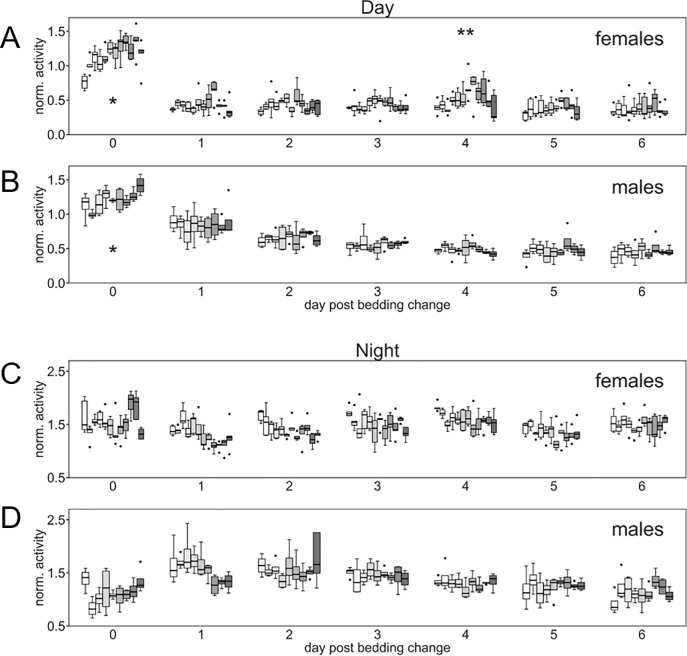
Day and night time activity of female and male mice at KI. Box plots (cages) showing normalized (see Material and methods) global activity (ordinate) in cages with female (A, B; n = 5) and male mice (C, D; n = 4) housed at KI, over several weeks (9–11), for each week day across a cage-change cycle (abscissa; 0 day of cage-change) for day-time (A, C) and night-time (B, D) activity, respectively. Asterisk denote day of cage-change while double asterisk indicates day of weighing. For both sexes there is a significant variation with weekday of the cage-changing cycle (p<0.001) and for the female but not the male mice across weeks (p<0.001), respectively. Analysis of effect of sexes and weekday on activity show that female mice show an increased activity during the day of the procedure (cage-change) while male mice show a significant impact by the procedure lasting several days both in day and night time activity. The difference of day and gender was significant at p<0.001.

At the KI-site male C57BL/6J were also monitored using the DVC system (Fig 6). The male mice housed at KI showed a significant variation in day and night time activity between week days (day factor p<0,001) but not across weeks (p>0.15). Closer inspection of day-time activity across weekdays during the cage-change cycle showed a distinct difference for the male mice with an effect of the cage-change (day 0) an impact both during day and night time (Fig 6C and 6D) fading off slowly across several days; this response pattern was not evident among the females which also displayed a significant variation across weeks (Fig 4).

**Fig 7:**
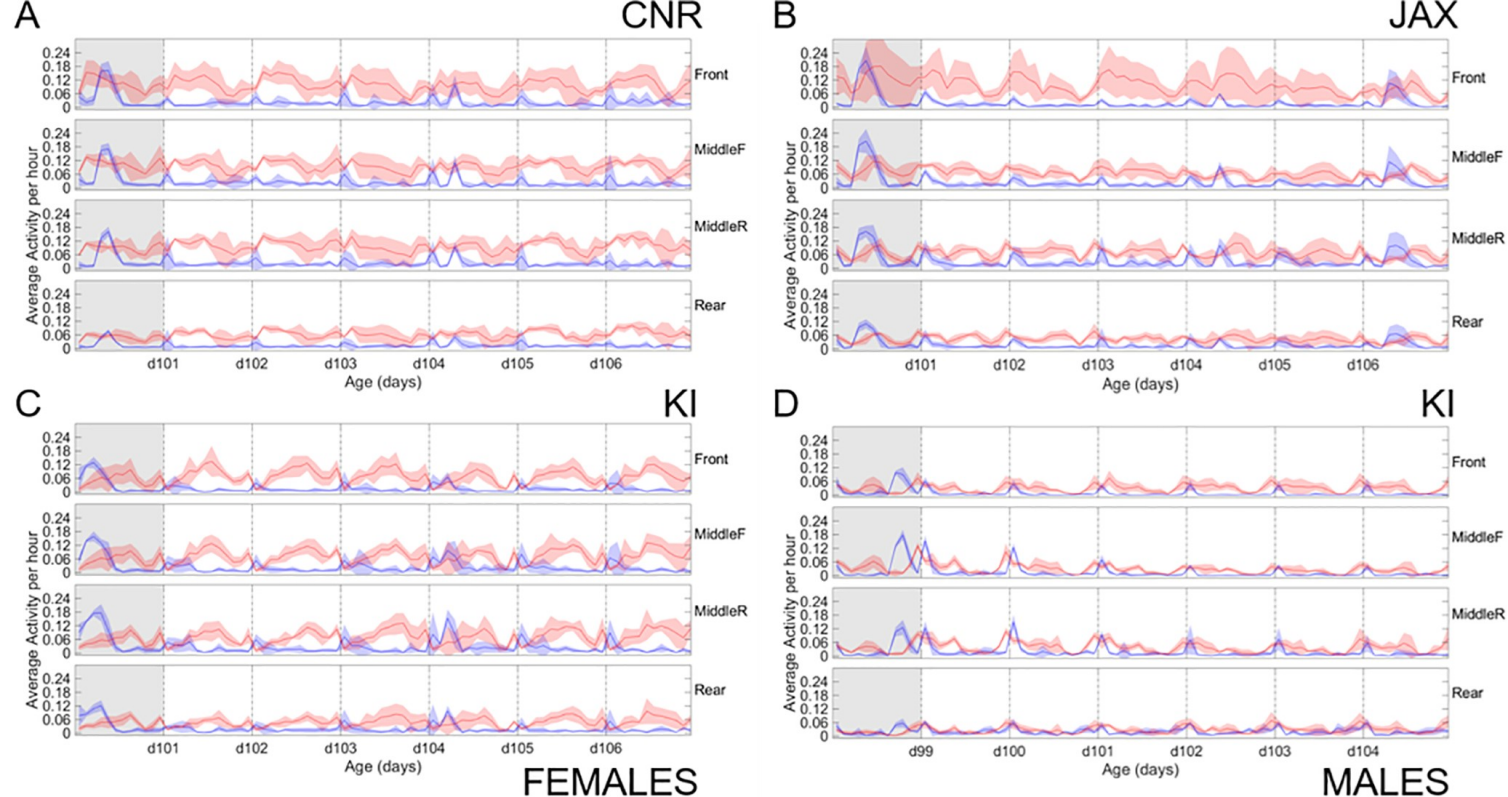
Distribution of activity during day and night time across the cage floor area at CNR, JAX and KI. Actigrams showing average activity in cages (A-D) during lights-on and lights-off in the front, middle-front, middle-rear and rear rows of electrodes at the three sites. Continuous activity (ordinate) data during one weeks (abscissa; age of the animals in days) from the cages with female mice at CNR (A), JAX (B), and KI (C) followed by the cages with male mice at KI (D). In A-D grey shaded area indicate day of cage-change. As in Fig 2 the blue trace (plotted as average per h based on activations per minute) is the average daytime activity (each bin starts at lights-on, and ends at lights-off) and shaded blue area denotes the standard deviation. The superimposed red trace is average activity during lights-off (each bin starts at lights-off and ends at lights-off; 12 hrs. phase shift from the daytime data) with the standard deviation indicated as red shaded area around the average trace. Note the overall lower level of activity recorded from rear area of the cage floor across sites and sexes. The level of activity day and night time is clearly lower in cages with male mice compared with cages holding female mice at KI. For further information, see text.

The use of the cage floor area was examined in some detail by grouping the activation data of each row of three electrodes into 4 floor areas (front, middle-front, middle-rear and rear, see Fig 1). The spatial distribution of day- and night time activity across the floor area showed a basic similarity between sites (Fig 7). Although the level of activity across day and night is lower in cages housing male mice than female mice, the distribution of activities across the cage floor shows no distinct sex difference (Fig 7C and 7D).

### Effects of procedures and recurring environmental events

**Fig 8:**
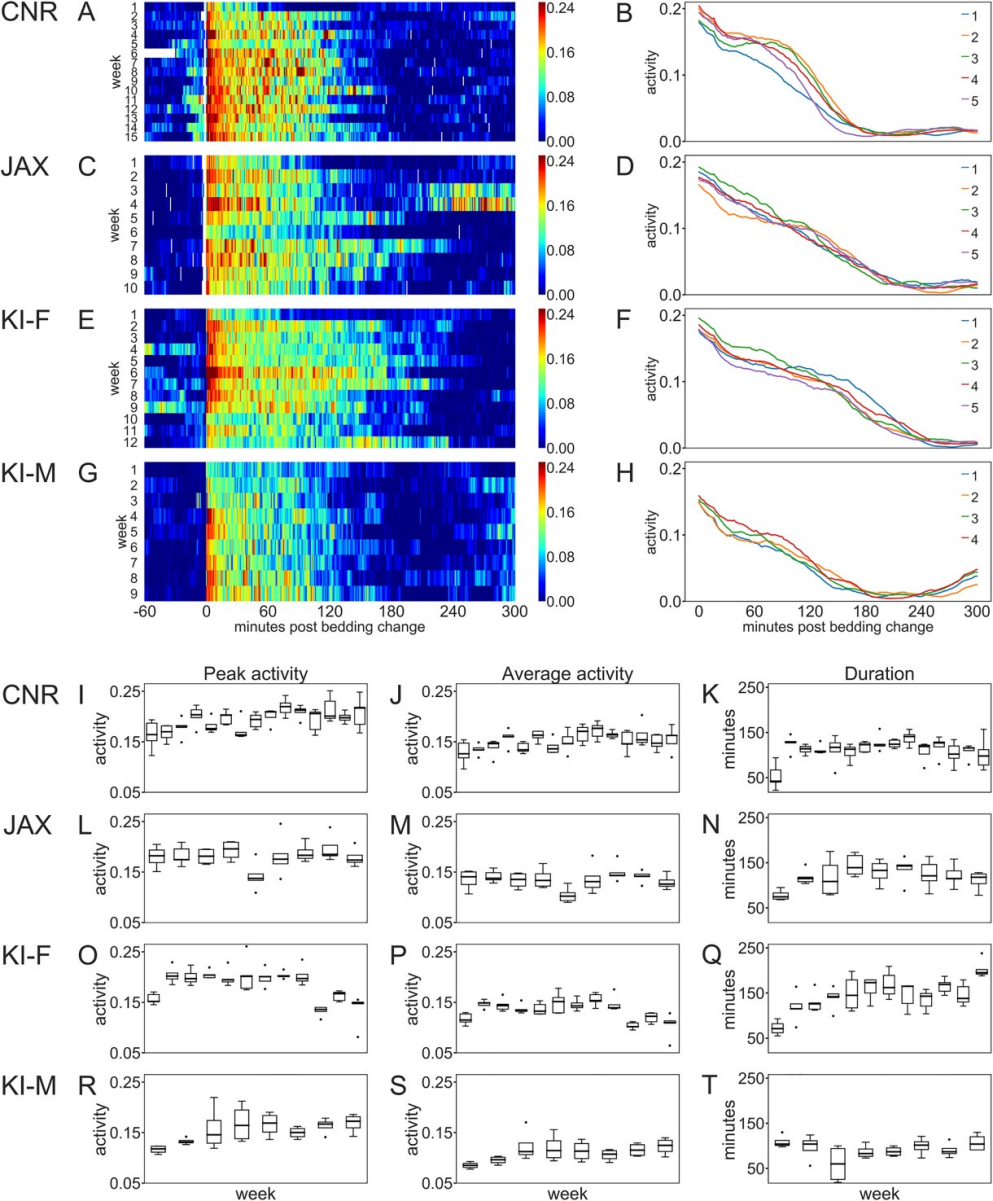
Impact of cage-change at CNR, JAX and KI. A, C, E and G Heat maps of single cages across multiple weeks (ordinate, each line represents a cage-change of sequential weeks; n = 9–15) showing the response to a cage-change (ordinate shows activity and abscissa time; the cage change start at time 0) in female mice (A, C, E) at the three sites and male mice at KI (G). Color coding according to scale to the right of each panel. B, D, F and H are plots of average response sampled across weeks for each cage (n = 5) with female mice at the three sites (B, D and F) and male mice housed at KI (H). Number of repetitive observations (weeks) are 15 at CNR, 3 at JAX, 12 for female mice at KI and 8 for male mice at KI. Peak- (I, L, O and R) and average activity (J, M, P and S) during the response duration (FWHM; see Material and methods) as well as the duration of the response (K, N, Q and T) were calculated from plots shown in B, D, F and H. Top row of panels show data collected from CNR, row two data from JAX, row three from KI and row four shows data from male mice housed at the KI site. Depending on the small number of observations at the JAX site it was excluded from the statistical calculations. The difference in average activity (p<0.01) and duration (p<0.001) of response (FWHM) to cage-change among female mice was significant between CNR and KI. Within sites there is a significant variation in peak-, average-activity and duration of response across weeks (p<0.01–0.001). Among male mice house at KI there was only a small difference in average activity across weeks in the response to cage-change (p<0.05). Peak- and average-activity, and response duration to cage-change were all significantly different between male and female mice housed at the KI site (p<0.01–0.001).

Procedures including handling, weighing and in particular, cage-change induce major alterations in activity patterns (Figs 2–4 and 6). Cage-change (Fig 8) induced a marked immediate effect lasting several hours among female mice and, thus, increasing general day time activity level. Such procedures underpin the day-to-day variance in activity at all three sites (see above).

We compared peak activity, average activity during the response and the duration (FWHM; see Material and methods) of the response to cage-change (Fig 8) at CNR and KI (too many missing data from the JAX site to enable inclusion in this comparison) and found that average-activity (higher at CNR; site factor p<0.01) and duration of response (shorter at CNR; site factor p<0.001) differed significantly between KI and CNR but also that there was a significant variation on-site between weeks in the peak, average activity response and duration of the response to the cage-change at both KI and CNR (week factor p<0.01–0.001). These metrics suggest that the response to cage-change are as expected dramatic across sites, and that the response is not due only to the cage-change *per se*, but also apparently site-specific factors and group dynamics of the animals inside the cage at CNR vs KI (JAX excluded from the analyses).

At the KI site we compared the response to cage-change of females and males using activity data normalized by average weekly activity. The analysis disclosed that males in contrast to females showed very little week-to-week variation (only in average activity; p = 0.045) and that direct comparison (factors sex and week) revealed a difference between males and females (week factor, p<0.01; sex factor, p<0,001) activity in the response to a cage-change (Fig 8E–8H and 8O-8T; see also Fig 6).

**Fig 9:**
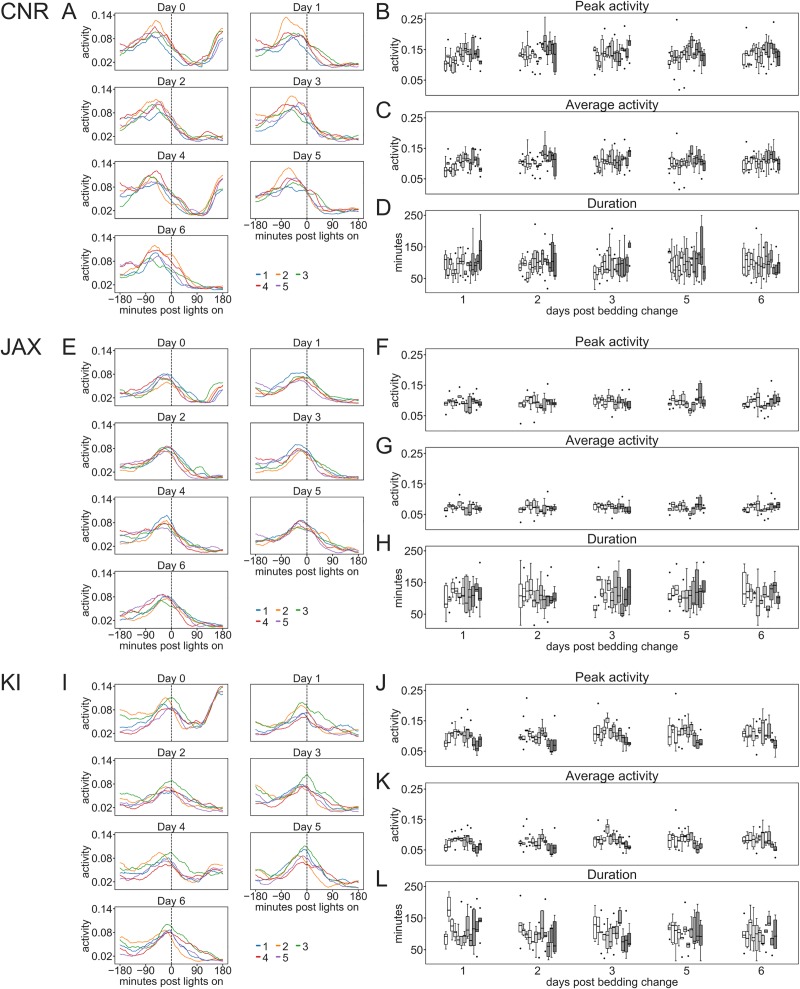
Impact by lights-on on activity at CNR, JAX and KI. A, E and I Plots of average response of each cage across 8–13 weeks during each day of a cage-change cycle (0 is day of cage-change) with female mice at CNR (A), JAX (B) and KI (C). Peak-(B, F and J) and average activity (C, G and K) during the response duration, and the duration (FWHM) of the response (D, H and L) were calculated from such plots. Among the female mice the peak and average activation response (site factor, p<0.001) but not the duration of the response (p>0.05) were significantly different between sites and across weeks of observations but not weekday of the cage-changing cycle. Within sites peak- activation varied across weeks (p<0.03–0.001) while only at the KI site there was a significant variation in both peak- and average activity response with day in the cage-changing cycle (p<0.01).

We used the same approach to analyze and measure the response to lights-on (Figs 2 and 9). This response differs from the cage-change (Fig 8) and lights-off responses (Fig 3) as it is accompanied by an increased activity both prior (~1h) to and following (~1h) lights-on (Figs 2 and 9). Peak- (site factor, 0<0.001) and average activity (site factor, p<0.001) during the response duration (FWHM) but not duration (site factor, p = 0.09) differed between sites. Within sites, there was a significant variation in peak activity across weeks (week factor, p<0.03–0.001) while a variation in peak- and average activity across week days was only evident at KI (factor week day, p<0.01–0.001).

**Fig 10:**
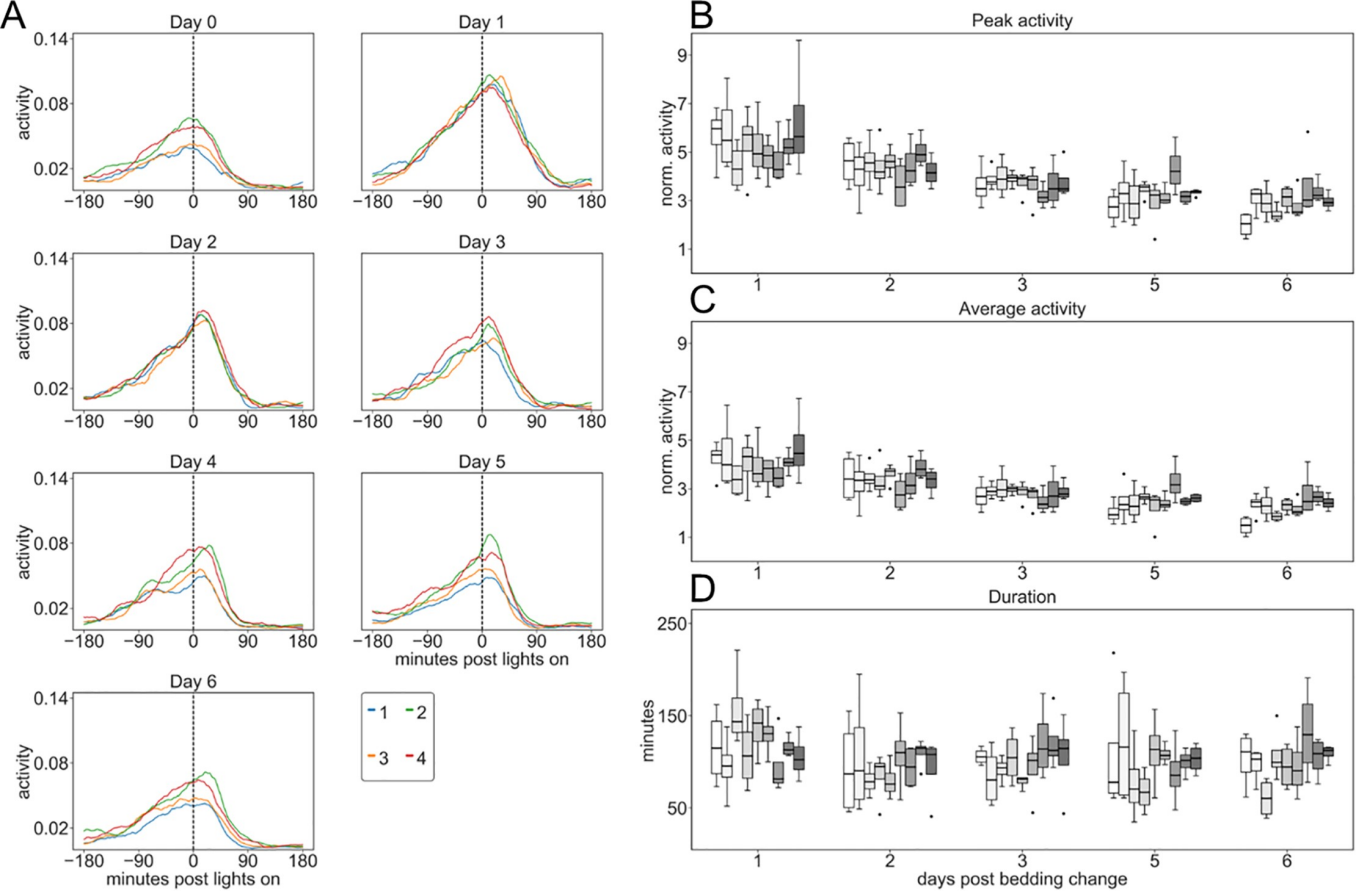
Impact by lights-on on activity of male mice at KI. (A) Plots of normalized (see Material and methods) average response of each day during a cage-change cycle (0 is day of cage-change) sampled across 8 weeks from cages with male mice at KI (see also Fig 9C for female mice at KI). Peak (B) and average (C) activity during the response duration and the duration (FWHM) of the response (D) of male mice (computed from plots shown in A) show that the peak- and average activity response varied with day in cage-change cycle (p<0.001) but not across weeks (p>0.10). While female mice (Fig 9J, 9K and 9L) show only small day-to-day variations following cage-change (p<0.01) and larger differences across weeks (p<0.001), the males show an increased response to lights-on the day after cage-change followed by a decreased response for 1–2 days until return to base-line response (day 5; p<0.001) which does not vary significantly across weeks (p>0.10). Statistical analyses revealed that both the peak- and average activity response is different between sexes (p<0.001) and that each sex has a different response across days post cage-change (p<0.001). In contrast, the duration does not show a distinct sex or weekday dependence.

In male cohort of cages held at KI (Fig 10; using normalized data, see Material and methods) the peak- and average activity response to lights on did not vary significantly across weeks (p>0.11) but with day of the cage changing cycle (day factor, p<0.001). Comparing males (Fig 10) with the females (Fig 9) at KI, show more intense response to lights-on the days that follow upon cage change cages with male mice. The difference between sexes was statistically significant (gender factor, p<0.001; day factor, 0.01–0.001; gender*day, p<0.001) for both peak- and average activity response but not for the duration of the response (p>0.14).

In summary, the general response to cage-change, light on and lights-off and other care procedures (weighing) are not site-specific for the female C57Bl/6J mice, while the magnitude of the response varies significantly between sites as well as between weeks on-site. Further, there appears to be a gender difference (experiments conducted at KI only) in the response to cage-change for the C57BL/6J males which also correlate with alterations in the response to lights-on, suggesting a carry-over effect of the impact of one procedure onto the response of other events.

## Discussion

### Objective of this work

It is clear that laboratory animals including inbred mice are highly complex, exhibiting adaptive behavior to a wide range of environmental situations or insults. Further, mice are regarded as a highly defined biological subject and their experimental reproducibility is often assumed as given. However, it is becoming more obvious that there can be considerable inconsistency in experimental outcome due to variable unscripted factors impacting the testing response(s) [19,26–29]. The development of automated high density, economic home cage monitoring systems offers the possibility that these variables can be explored, understood and subsequently controlled. In the past 10 years such systems are beginning to be developed and used, and their potential benefits are a subject of great interest to the community (reviewed in [16]; [6]). There are a host of advantages such systems could provide with their capacity to deliver continuous, economically scalable, home cage and unbiased surveillance (24/7) data. Such systems are complementary to the current daily spot welfare checks, augmenting these observations, providing quantitative unbiased data and increased awareness of reproducibility.

### Unbiased data collection in simple scalable systems

We here describe the DVC^TM^ system, which is capable of measuring mouse activity inside standard IVC housing home cages unobtrusively. The DVC^TM^ system is based on the detection and tracking of electrical capacitance perturbations occurring across the cage floor via the use of a capacitive-based electrode array. The electrodes are installed externally and under each IVC cage floor collecting data every 250 msec, 24/7. These data enable temporal and spatial separation of cage activities generated cumulatively by all mice housed in the cage that occurs on the cage floor. Signal analysis using the first-order time difference have proven useful in providing an initial descriptive data on life inside the home cage, and represents our primary attempts in data analysis from the DVC^TM^ system (see also [30]). We envision that upon gaining more experience in elaborating these data that we will be able to extract a richer set of activity metrics in real time.

A variety of technical solutions to enable home cage monitoring are currently being tested/used including video tracking, infrared light beams, RFID technology and here, capacitive-based sensing technology, as well as combinations of these technical solutions. Each of these approaches have advantages and disadvantages (discussed in [6]), here we will briefly outline these for the capacitive-based technology as implemented in the DVC^TM^. The DVC^TM^ platform can be fully integrated in standard GM500 IVC rack. The system is comparatively economic, enabling large scale deployment and real-time access to data. Further, the demands on data storage and CPU power are modest compared to, for example video tracking. The exploration of how the raw data from the DVC^TM^ sensor can be used is still early in development and here we only used (electrode) *activation* however, we foresee that detection of animals resting and direction of movement will be possible. Further, when only one or two animals are housed in the cage, more sophisticated metrics such as trajectory tracking, velocity as well as social proximity interaction should be possible to obtain with reasonable accuracy. Further, such systems can be used to accurately monitor changes in behavior in groups of animals subjected to, for example different drug testing regimes e.g. mice with ALS [31].

### Rhythmicity of activity across day and night

Our data shows that cage life in terms of activity for both male and female C57BL/6J follows a rhythmicity in activity across night and day (diurnal pattern) with rest during lights on [9,10,13,14,16,32,33]. With lights-off at night, activity rapidly increases and remains at a variable but high level for two-thirds of this period. Late, 6–8 hrs. after lights off, activity declines almost to day time resting pattern. Preceding lights on, activity increases sharply, with a further activity burst occurring around lights-on and continuing for around 1h after light-on (idem). Thus, this response is not elicited by the light stimulus itself, but may be due the diurnal rhythm anticipating day break, signaling of danger intrinsic to this species. These basic pattern of activity across day and night were recorded independently at all three sites participating in this study and is thus not a local establishment phenomenon and also validated for both male and female mice at KI. This diurnal rhythm of activity is similar to those presented for group-held C57BL/6J using RFID based technology recordings ([16]) and also with rhythmicity of activity recorded across day and night for single-housed C57Bl/6J [10,13,14,32] suggesting a robustness of this phenotype trait and an activity pattern during night time that distinguishes this strain from several other mouse strains [13,14,19,29,34].

An important aspect of this work is showing that access to continuous recording output from the electrodes at and across sites enables unobtrusive capturing of details of in-cage life. Thus the data show that although the diurnal pattern of activity replicates across sites, significant variances occurs within and between sites, such variances are evident as differences between records from sites and across weeks within sites, reflecting unscripted, perhaps subtle, differences between environments at sites and other variances due to differences in group dynamics of the animals in the cages (see also [19,29,30]). For example, the over-all higher frequency of activations day and night recorded at CNR remains unclear. Post hoc analysis revealed that the vivarium room at CNR was 15.5 m^2^ and only technicians (2–3) involved in the multicenter study had access to this room. In contrast, the vivarium at KI (floor area 20 m^2^) and JAX (117 m^2^), respectively, are larger and several research groups were active during the weekdays in these rooms (Table 3). Also normal light levels at the front of the cage varied between sites being lower at JAX than CNR and the lowest at KI (idem).

The variability across weeks within sites among female mice (Fig 4) may relate to activity alterations caused by the estrus cycle [35–37]. It cannot be ruled out that some calendar weeks held two proestrous increase in activity and other weeks only one peak, causing the week-to-week variability that could not be detected among the male C57Bl/6J mice housed at KI.

A second observed difference was the gradual rather than sudden increase in activity noted at KI in response to lights-off while at both CNR and JAX this change occurred more or less as an immediate response to lights-off (Fig 2) consistent with previous published data of single and group held C57Bl/6J [16,29,32]. A third difference is that the peak of response to lights-on precedes the actual event at CNR but not at the JAX and KI sites, however, such differences are evident also in studies of single housed C57Bl/6J (idem). The cause of these two further behavioral differences remains elusive; however it may be again linked to local site factors as light levels at the cage and the parallel activities in the holding quarters at JAX and KI impacting the mouse environment (Table 3).

As shown here, response to stimuli that may act as stressors, differ both in magnitude and duration due to group dynamics inside the cage, the holding site and, importantly, gender of the animals. To proper compare across gender with different cage conditions (Table 2), we used a normalized activity metric since we wanted to measure the response to cage change with respect to a baseline activity, which is captured here by the average weekly activity. Inspecting raw data gathered here (see Fig 7) suggests that female mice may be more active than male mice of this strain which would be consistent with other observations on gender difference in explorative behavior of this mouse strain [38] and may relate to the estrous cycling of the females (see however [37]).

Combined, the here observed differences between sites and within sites clearly underscore the need for further exploration of environmental parameters impacting mouse activity in-cage.

In a long term the type of data reported here will assist in refining husbandry, including seasonal variation as well as in monitoring behaviors during animal testing, paving the way for improvements of testing reproducibility. One caveat which needs to be considered here is that these data are derive from a single strain of mice, reflects only group cage activity and, activity on the cage floor only.

### Impact by cage change

Of crucial importance, our data suggests that procedures involving handling or transfer of the animals (e.g. cage-changes) disrupts their basic activity pattern and induces changes that appear to impact over an extended period of time (cf. Figs 2, 3 and 6). Males, observed at the KI site, showed a clearly more long-lasting impact on average day-time activity following cage-change than did female mice. The reason for this difference needs to be investigated in detail but may be caused by the more complex re-establishment of hierarchy and territory control among males than females. As shown here, a cage-change may in male but not female C57BL/6J mice also influence other responses, for example, increasing activity at lights-on for days following the cage-change (Figs 9 and 10). As such, such perturbations should be assessed and/or built into an experimental design. We believe the observations presented here will be valuable in future design of animal testing and animal welfare surveillance and show that mice represent a complex subject and that needs careful considerations when designing experiments.

### Concluding remarks

Automated home-cage 24/7 monitoring systems may thus assist with detection of behavioral alterations, indicating environmental impactors, illness or the development of a deviating phenotype, or the response to experimental testing. Such sensitive systems also open for behavioral analyses of different functional domains of the brain to be conducted in the home-cage environment without handling by an experimenter ([21]). As such, home cage monitoring can facilitate the implementation of humane end points and also be an important tool in refinement of husbandry to improve animal welfare including breeding performance. As noted here, site-specific differences were present despite efforts to make conditions as similar as possible and post hoc analysis could not with certainty pin-point the reason(s) for these discrepancies, suggesting that the granularity of protocols and housing conditions need further refinements. As such information becomes more generally available its will point towards the importance of harmonizing husbandry and procedure protocols across laboratories to promote reproducibility and making animals experiments more powerful, whilst respecting the 3R’s.

## Supporting information

S1 File (PDF)

S1 Fig Response to lights on. The response to lights on is identified as follows (see S1 Fig):
• Smooth the minute-based activity time series (averaged across all 12 electrodes) with a low pass filter (moving average of 30 minutes)
• Find the peak of the time series (within +/- 3 hours from lights on)
• Find contiguous blocks of minutes whose activity is larger than half of the peak
• If there are less than 5 minutes between two blocks, consider them as a single block
• Pick the block which contains the maximum value (block 2 in the example in S1 Fig)
• Set the response duration as the distance between the extrema of the identified block (points C and D in the example in S1 Fig).
(TIFF)

S2 Fig Heat map for cages with male mice with bi-weekly cage change. Heat maps showing average global activity of four cages with male C57B/6J mice, kept 5 to a cage, during 4 consecutive weeks (day 1–28); conversion of activity to color according to scale to the right. The basic pattern of day and night time activity levels are the same as for the cages with weekly cage-change (cf. S2 Fig and Fig 2). Following the cage-change day the day and night activity pattern reach a level that is essentially maintained as a basal undisrupted activity patter until next cage-change. Cage-change day 2 and day 16 have been indicated with an asterisk and white vertical line indicates transition to night time while left and right border of the heath map correspond to day break.
(TIF)
