# Supplementary material for: Towards large scale automated cage monitoring – Diurnal rhythm and impact of interventions on in-cage activity of C57BL/6J mice recorded 24/7 with a non-disrupting capacitive-based technique
Source: PLoS One. 2019 Feb 4;14(2):e0211063. doi: 10.1371/journal.pone.0211063 (PMC6361443; doi:10.1371/journal.pone.0211063)
Supplement: S1 File — (PDF) [file pone.0211063.s001.pdf]

Supporting information: Towards Large Scale Automated Cage Monitoring

Towards Large Scale Automated Cage Monitoring – Diurnal rhythm and impact of interventions on in-cage activity of C57BL/6J mice recorded 24/7 with a non-disrupting capacitive-based technique

K. Pernold, F. Iannello, B. E. Low, M. Rigamonti, G. Rosati, F. Scavizzi , J. Wang, M. Raspa, M. V. Wiles, and B. Ulfhake

S1 Fig Response to lights on

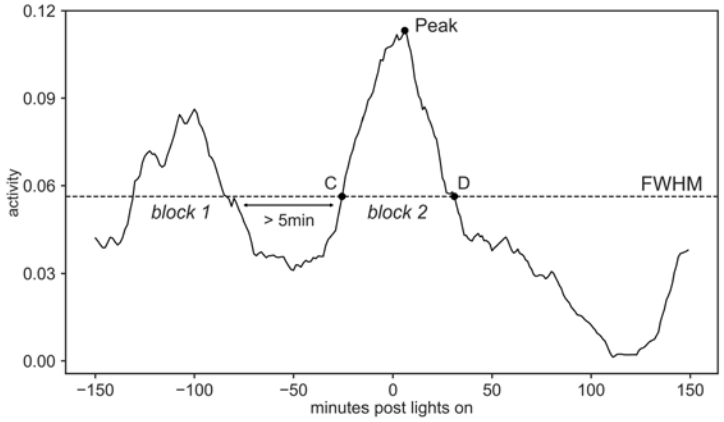

The response to lights on is identified as follows (see Fig. S1):

- Smooth the minute-based activity time series (averaged across all 12 electrodes) with a low pass filter (moving average of 30 minutes)
- Find the peak of the time series (within +/- 3 hours from lights on)
- Find contiguous blocks of minutes whose activity is larger than half of the peak
- If there are less than 5 minutes between two blocks, consider them as a single block
- Pick the block which contains the maximum value (block 2 in the example in Fig. S1)
- Set the response duration as the distance between the extrema of the identified block (points C and D in the example in Fig. S1)

S2 Fig Heat map for cages with male mice with bi-weekly cage change

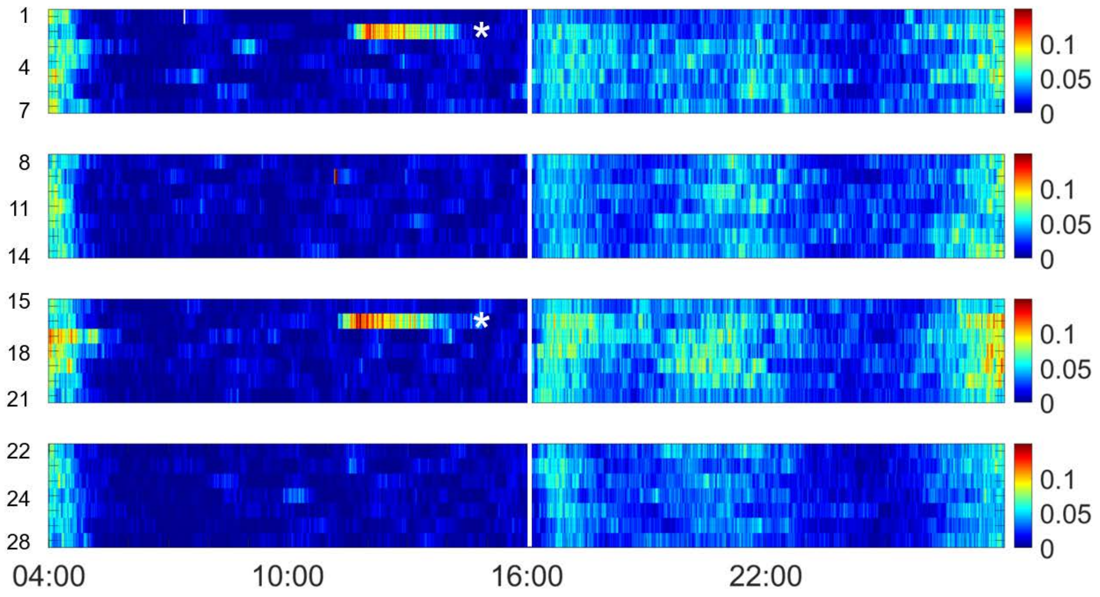

Heat maps showing average global activity of four cages with male C57B/6J mice, kept 5 to a cage, during 4 consecutive weeks (day 1-28); conversion of activity to color according to scale to the right. The basic pattern of day and night time activity levels are the same as for the cages with weekly cage-change (cf. S2 Figand Fig 2). Following the cage-change day the day and night activity pattern reach a level that is

essentially maintained as a basal undisrupted activity patter until next cage-change. Cage-change day 2 and day 16 have been indicated with an asterisk and white vertical line indicates transition to night time while left and right border of the heath map correspond to day break.

Digital ventilated cage^{TM} (DVC^{TM})

Digital ventilated cage (DVC^{TM}) is a commercially available system designed to collect information from individual ventilated cages (IVC) directly at rack level. DVC^{TM} system builds up on the top of a standard IVC rack by resorting to a set of sensors, which are able monitor cage conditions as well as animal activity. All sensors are placed outside the cage and do not require any human intervention to collect data, nor impacting conventional IVC cage operations. The key DVC^{TM} sensing component is an electronic board installed below each IVC cage and mechanically connected to the rack. This electronic board is composed of 12 planar electrodes connected to an integrated circuit (*proximity sensor*) that continuously measures their electrical capacitance. Since electrical capacitance is generally influenced by the matter present in each electrode's surrounding, the capacitance measured by the proximity sensor depends on the presence of, e.g., bedding material, water, animals, etc. By properly tracking and analyzing capacitance variation over time, it is possible to detect animal activity as well as bedding humidity condition (due to e.g., latrine). The proximity sensor measures the electrical capacitance of each of the 12 electrodes 4 times per second (i.e., every 0.25 seconds), thus generating a total of 48 samples per second per board (cage). In addition to the 12-electrodes board, the DVC^{TM} system also embeds IR sensors at each cage position (connected to the rack laterally with respect to the cage) to monitor the presence of food and water.

All electronics components of DVC^{TM} are connected via wires to a dedicated computer, referred to as *DVC^{TM} master*, which provides both power and data connection. The DVC^{TM} master collects raw data coming from all cage positions, with the option of both transferring them directly to a web-based software application or to a dedicated storage device for later data processing purposes.

It is worth to mention that, DVC^{TM} is a big-data oriented system that is designed to collect, store and process data from thousands of cages simultaneously (i.e., *high throughput*). In addition, DVC^{TM} system requires neither manual intervention to set up the sensing infrastructure, as this is inherently connected to the rack and always on, nor maintenance or removal of components as these have been designed to be safely sanitized and/or inserted into autoclaves while still connected to the rack.

Computation of the activity metric

For the scope of this study, it was decided to measure animal activity in terms of perturbation of electrode capacitance over time. As mentioned above, the rational is that, when an animal moves closer to an electrode or away, the measured capacitance varies accordingly, whereas if no movements occur, no capacitance changes (or very small due to noise) are recorded. The DVC^{TM} animal activity algorithm used throughout the paper translates these observations into metrics as follows. For any given DVC^{TM} board corresponding to a TMspecific cage position, let $c_k(t)$ be the measure of the kth electrode at discrete time t, with $k \in \{1,2,\ldots,12\}$ and $t \in \{0,1,2,\ldots\}$. Recall that, a sample is collected every 0.25 secs. Capacitance perturbations are then evaluated via one-step finite difference $d_k(t) = c_k(t) - c_k(t-1)$, with $t > 0$. The activity indication at the kth electrode at time t is determined by comparing the finite difference $d_k(t)$ against a fixed threshold λ as follows $a_k(t) = 1(|d_k(t)| \geq \lambda)$, where $1(E)$ is an indicator function for event E, $1(E) = 1$ if event E is true and $1(E) = 0$ if event E is false. The threshold λ is conveniently selected based on noise magnitude considerations and it is assumed fixed and equal to $\lambda = 2$ throughout the whole paper. Depending on the needs, a summarizing activity metric can be built up upon $d_k(t)$ by summing up activations within a given time interval, and possibly by averaging across a set of electrodes. More

specifically, let t_1 and t_2 be the starting and ending time of the required time interval, and let S be a set of electrodes of interest, then the average activity metric is calculated as:

$$A_S(t_1, t_2) = \frac{1}{(t_2 - t_1)|S|} \sum_{k \in S} \sum_{l=t_1}^{t_2} a_k(l).$$

Note that, activity metric $A_S(t_1, t_2)$ is mainly used in this paper to measure activity within 1 minute intervals for each minute of the day (t_1, t_2 are selected to cover 1 minute), or for lights ON/lights OFF periods (where t_1, t_2 cover, e.g., 12 hours). Moreover, the set of electrodes S is used to indicate either the whole cage (all 12 electrodes, i.e., $= \{1,2,\dots,12\}$), or electrodes in the front of the cage ($S = \{10,11,12\}$), middle ($S = \{4,5,6,7,8,9\}$) and rear ($S = \{1,2,3\}$). It is important to emphasize that DVC can measure overall activity for the mice within a cage, without discerning each animal individually.

Statistical analyses

Data were processed through scripts generated in either R, MATLABTM or PythonTM. We used the rank-based analysis of variance-type statistic (ATS) to test differences across sites or sexes, weeks and days (23). This is a non-parametric test for longitudinal data that does not require strong assumptions as the Repeated Measures ANOVA (which we found violated in some cases). Analyses were implemented using the nparLD package in R statistical software (24). We considered cages as subjects, weeks and days post bedding change as within-subject factors ("sub-plot" repeated factors), site and sex as between-subject factors ("whole-plot" factors). According to authors' terminology (24), we used four different tests in the paper: LD-F1 (one repeated factor: week), LD-F2 (two repeated factors: week and days), F1-LD-F1 (site or sex as the whole-plot factor and week as the repeated one), F1-LD-F2 (site or sex as the whole-plot factor and weeks and days as the repeated ones).

(for references see full paper)

Husbandry protocol multicenter study

The protocol of the 3-site study:
Welfare check using Tecniplast DVC system – protocol draft (weeks 5:46-6:10)
5th Aug 2015 JAX(mvw)+CNR and KI
Objective
To test if the DVC/sensor caging system can be used to detect behavioral changes due to disease or phenotype.
Daily Welfare Checks: i.e. food, water, flooding, live/dead/distressed
Regarding feed/water/stress and other site differences: the experiments are essentially self-contained – i.e. local feed, water and their impact on the mice are not strictly relevant as we will be comparing to control animals within the same cage.
Using Tecniplast MiniRack
Animal model:
C57BL/6J
Experiment will use:
75 females and 20 males.
Mice to be sent out at ~4 (female) and ~6 (male) weeks of age
JAX first, CNR and KI second in parallel
JAX will put mice into DVC cages at 5 weeks of age
At KI it will be ~ 9 weeks of age
CNR will start at week 5-6 (depending on the logistic issue)
Numbers of animals
Females = 5 mice per cage, 5 cages; 5x5 females; 25 per site
Male = 5 mice per cage, 4 cages; 5x4 males; 20 at KI
MiniRack configuration:

Control	Test 2	Test 1	Test 1	Control
Test 1	Control	Test 2	Control	Test 2
Test 2	Test 1	Control	Test 2	Test 1

BEDDING:

Quantity: 200g. Type: corn cob 1/4 inch

KI-M: 100g aspen chips

CAGE CHANGE INTERVAL:

Every week, fixed day: every Thursday

KI-M: Every week, fixed day: every Tuesday between 11-12AM. Including weighing.

BIWEEKLY WEIGHING:

Fixed day: every Monday and Thursday

ENRICHMENT:

Tecniplast Mouse House that stays in cage

NESTING MATERIAL:

One piece of Nestlet, will be replaced at cage change

KI-M: “sizzle nest”, transferred at cage change

WATER:

CNR is the only one with chlorinated water and then acidified. JAX and KI only acidified.

FOOD:

JAX: LabDiet 5K52 autoclaved and we believe “extruded”

CNR: SDS RM3 Food -irradiated

KI: SDS RM3 Food-irradiated

LENGTH OF WATER BOTTLE NIPPLE:

Normal current Water Bottle length.

ANIMAL TAGGING:

In order to individually identify animals (weight, behavior, etc.) , these must be tagged. Ear notches is the preferred method.

Daily checks and EXPERIMENTAL ENDPOINT:

Daily welfare checks as normal –

At weighing look for symptoms on all mice

loss of weight

loss of condition

Experiments should be completed by 24 weeks of age
