# Supplementary figures and images for: Towards large scale automated cage monitoring – Diurnal rhythm and impact of interventions on in-cage activity of C57BL/6J mice recorded 24/7 with a non-disrupting capacitive-based technique
Source: PLoS One. 2019 Feb 4;14(2):e0211063. doi: 10.1371/journal.pone.0211063 (PMC6361443; doi:10.1371/journal.pone.0211063)

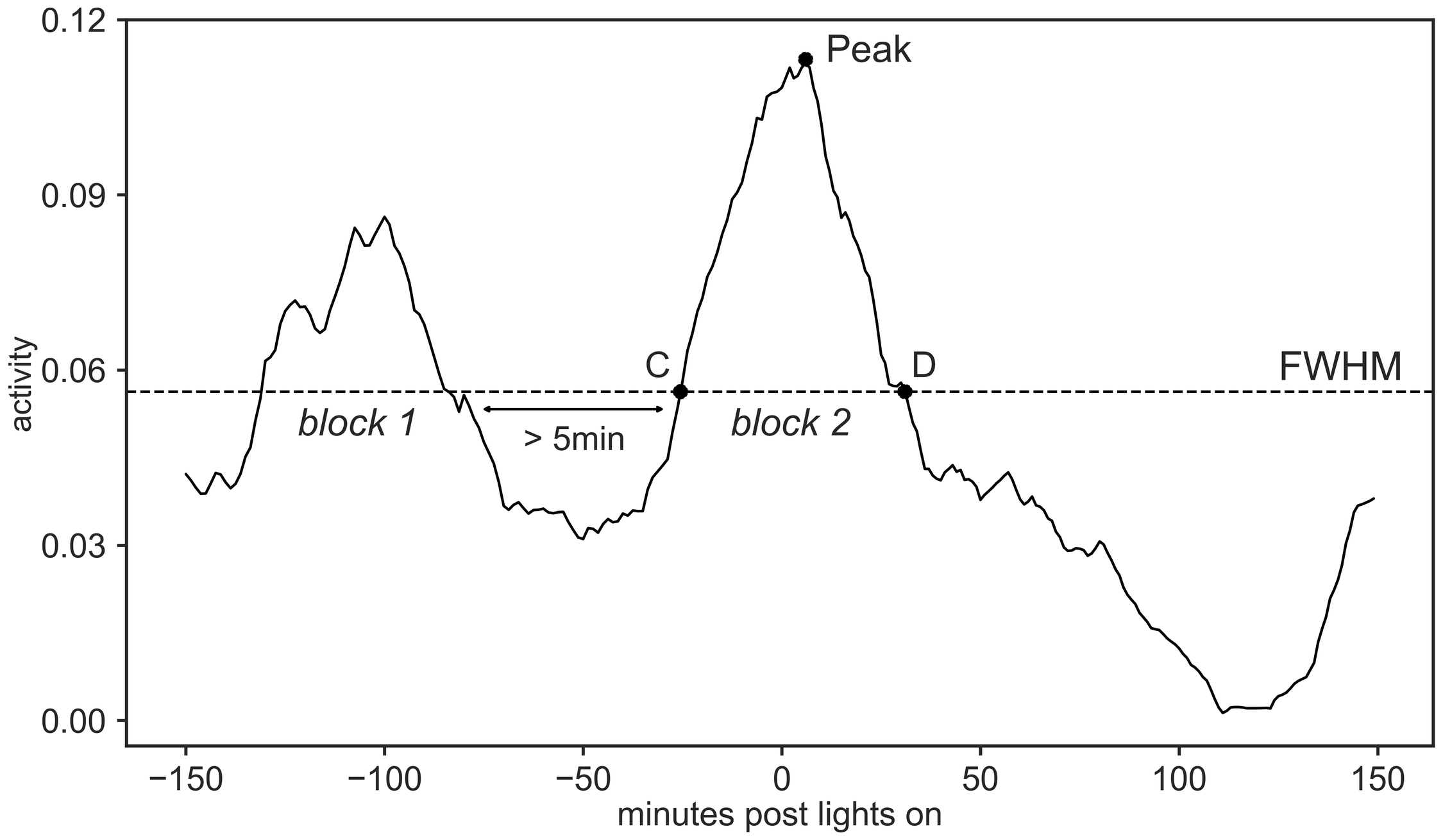

Supplement: S1 Fig — The response to lights on is identified as follows (see S1 Fig): • Smooth the minute-based activity time series (averaged across all 12 electrodes) with a low pass filter (moving average of 30 minutes) • Find the peak of the time series (within +/- 3 hours from lights on) • Find contiguous blocks of minutes whose activity is larger than half of the peak • If there are less than 5 minutes between two blocks, consider them as a single block • Pick the block which contains the maximum value (block 2 in the example in S1 Fig) • Set the response duration as the distance between the extrema of the identified block (points C and D in the example in S1 Fig). (TIFF) [file pone.0211063.s002.tiff]

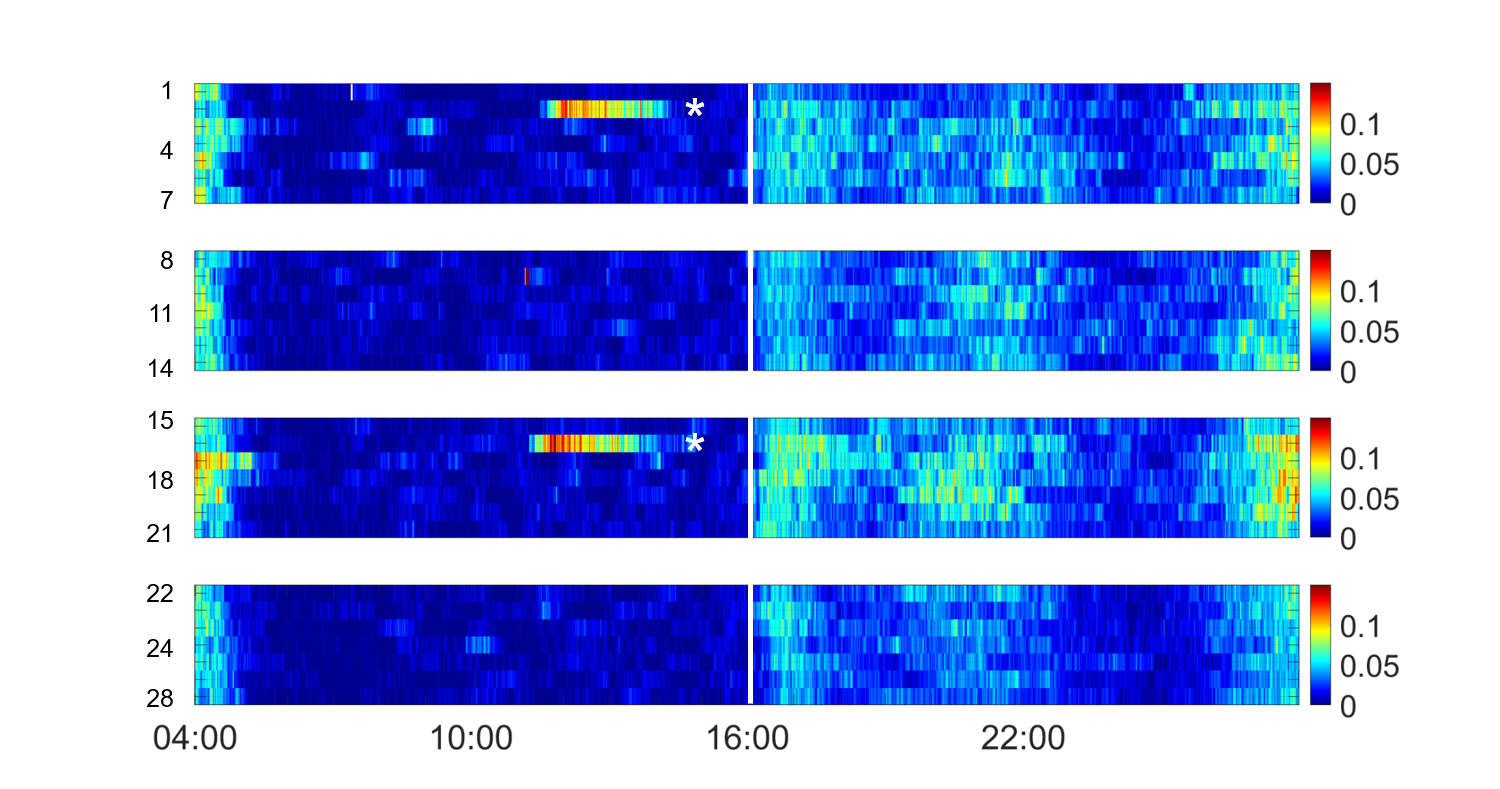

Supplement: S2 Fig — Heat maps showing average global activity of four cages with male C57B/6J mice, kept 5 to a cage, during 4 consecutive weeks (day 1–28); conversion of activity to color according to scale to the right. The basic pattern of day and night time activity levels are the same as for the cages with weekly cage-change (cf. S2 Fig and Fig 2). Following the cage-change day the day and night activity pattern reach a level that is essentially maintained as a basal undisrupted activity patter until next cage-change. Cage-change day 2 and day 16 have been indicated with an asterisk and white vertical line indicates transition to night time while left and right border of the heath map correspond to day break. (TIF) [file pone.0211063.s003.tif]
